# The Impact of Fluorination on the Design of Histone Deacetylase Inhibitors

**DOI:** 10.3390/molecules28041973

**Published:** 2023-02-19

**Authors:** Duong Tien Anh, Nguyen Hai Nam, Brigitte Kircher, Daniel Baecker

**Affiliations:** 1 Department of Pharmaceutical Chemistry, Hanoi University of Pharmacy, 13-15 Le Thanh Tong, Hanoi 10000, Vietnam; 2 Immunobiology and Stem Cell Laboratory, Department of Internal Medicine V (Hematology and Oncology), Medical University Innsbruck, Anichstraße 35, 6020 Innsbruck, Austria; 3 Tyrolean Cancer Research Institute, Innrain 66, 6020 Innsbruck, Austria; 4 Department of Pharmaceutical and Medicinal Chemistry, Institute of Pharmacy, University of Greifswald, Friedrich-Ludwig-Jahn-Straße 17, 17489 Greifswald, Germany

**Keywords:** fluorination, fluorine, fluorine-18, histone deacetylase (HDAC), histone deacetylase inhibitors (HDACis), positron emission tomography (PET), potency, selectivity, suberoylanilide hydroxamic acid (SAHA), vorinostat

## Abstract

In recent years, histone deacetylases (HDACs) have emerged as promising targets in the treatment of cancer. The approach is to inhibit HDACs with drugs known as HDAC inhibitors (HDACis). Such HDACis are broadly classified according to their chemical structure, e.g., hydroxamic acids, benzamides, thiols, short-chain fatty acids, and cyclic peptides. Fluorination plays an important role in the medicinal–chemical design of new active representatives. As a result of the introduction of fluorine into the chemical structure, parameters such as potency or selectivity towards isoforms of HDACs can be increased. However, the impact of fluorination cannot always be clearly deduced. Nevertheless, a change in lipophilicity and, hence, solubility, as well as permeability, can influence the potency. The selectivity towards certain HDACs isoforms can be explained by special interactions of fluorinated compounds with the structure of the slightly different enzymes. Another aspect is that for a more detailed investigation of newly synthesized fluorine-containing active compounds, fluorination is often used for the purpose of labeling. Aside from the isotope ^19^F, which can be detected by nuclear magnetic resonance spectroscopy, the positron emission tomography of ^18^F plays a major role. However, to our best knowledge, a survey of the general effects of fluorination on HDACis development is lacking in the literature to date. Therefore, the aim of this review is to highlight the introduction of fluorine in the course of chemical synthesis and the impact on biological activity, using selected examples of recently developed fluorinated HDACis.

## 1. Introduction

In recent decades, progress has been made towards the faster diagnosis of cancer, for example, by multimodal imaging [1], focusing on particular biomarkers and subsequent analysis using omics technologies [2,3]. Distinct treatment approaches—apart from classical chemotherapy using anticancer drugs—such as through sophisticated stem cell transplantation [4,5] are resulting in novel opportunities in cancer treatment. Despite these achievements, cancer remains one of the leading causes of death worldwide [6].

Intense research into the pathogenesis of cancer generally improves our knowledge of the disease. However, the current advances in understanding the pathogenesis of cancer are only a small step forward. Indeed, many aspects are still completely unclear and a comprehensive understanding is needed for successful prevention and targeted treatment [7].

Further challenges include circumventing the development of resistance and achieving the best possible selectivity against cancer cells. Therefore, it is important to make smart structural modifications to existing anticancer drugs, or even better, to find auspicious targets that can be addressed by novel classes of bioactive compounds. In this context, various cancer-associated enzymes are coming into focus to serve as potential targets for antitumor therapeutics [8]. One promising representative among them are histone deacetylases (HDACs) [9].

Histones are the substrates of HDACs. After the deacetylation of acetylated lysine, the amino acid located at the *N*-terminal end of the histones is again positively charged. This is accompanied by an increased affinity for the negatively charged phosphate backbone of the DNA, ultimately resulting in a downregulation of its transcription. The dysregulation of gene expression, which is actually mediated by HDACs, is known to occur in a variety of cancers [10]. The abnormal activation of HDACs can be targeted by HDAC inhibitors (HDACis).

**Figure 1:**
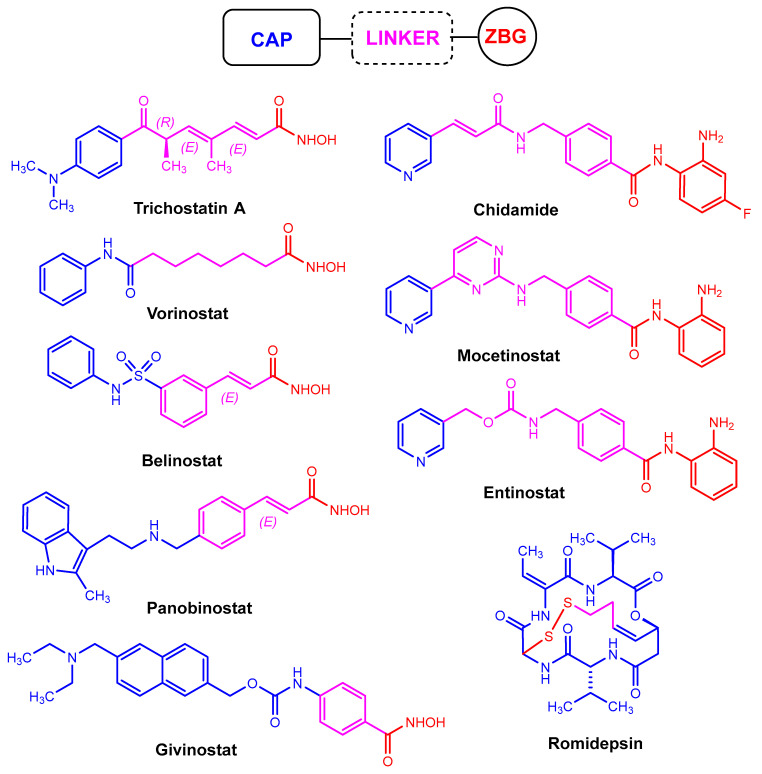
Chemical structures of some HDACis considering the pharmacophore consisting of cap, linker, and zinc-binding group (ZBG).

Probably the first HDACi to be discovered is trichostatin A (Figure 1), which is currently undergoing preclinical studies. However, some HDACis have already been approved by the United States Food and Drug Administration (FDA) for the treatment of various types of cancer. Some examples include the cyclic peptide romidepsin, and the hydroxamates vorinostat (also known as SAHA—suberoylanilide hydroxamic acid), belinostat, and panobinostat (Figure 1) [11]. In addition, some interesting synergistic properties were also discovered when HDACis were combined with established anticancer drugs [12]. All these encouraging features ensure that new HDACis are constantly being developed.

Our previous research on the design of HDACis involved derivatives bearing different heterocyclic cores, such as compounds based on quinazoline [13,14,15,16,17], indirubin [18], as well as 1,3-oxazole and 1,3-thiazole [19]. The use of heterocycles plays an important role in medicinal chemistry [20]. Within the concept of bioisosterism, heterocycles can be used to optimize drugs, for example, regarding pharmacokinetic and toxicological properties, efficacy, and selectivity [21].

Fluorination is another very common method of fine-tuning drug molecules [22,23,24]. This concerns, for instance, the development of glycomimetic drugs [25], metal complexes [26], and nucleosides [27], with all of these exerting also anticancer activity. The introduction of fluorine substituents in antitumor active compounds is performed for various reasons, such as bioanalytical labeling, in order to increase selectivity [28] or regulate the mechanism of cell death [29]. Fluorination is also a common method in the design of HDACis. However, to the best of our knowledge, it has no explicit focus in the literature thus far. Therefore, the aim of this review is to gain insight into the purpose and influence of fluorination in the recent development of HDACis.

## 2. Introduction of the Chemical Modification Generating Fluorination in HDACis

In the last decade, many HDACis with different core structures have been discovered, including short-chain fatty acids (e.g., phenylbutyric acid, valproic acid), hydroxamic acids (e.g., SAHA or suberoylanilide hydroxamic acid), benzamides, and acyclic peptides (e.g., depsipeptide). Nevertheless, they are subject to a very similar structural setup. The established pharmacophore comprises a cap and a zinc-binding group (ZBG), both of which are connected by a linker that occupies the binding channel (Figure 1) [30]. The cap interacts with the pockets of zinc-dependent HDAC, while the zinc-binding domain chelates zinc [31].

Several representatives of HDACis have undergone different phases of clinical trials for numerous types of cancer [32]. With the efforts to find new anticancer drugs, many studies have been carried out to search for novel potential HDACis, while fluorination plays a significant role. The different processes of introducing fluoro groups into these HDACis vary regarding the synthetic pathways, methods, and reaction conditions. This comprehensive work aims to summarize the progress that has been made in recent years on the fluorination of HDACis as anticancer agents.

### 2.1. Hydroxamic Acids

Hydroxamic acids are the best studied class of HDACis. To date, three of the four agents approved by the FDA with a confirmed HDAC-mediated mechanism of action for treatment in the clinic are hydroxamic acids. The representatives of this subclass are also the most studied group of fluorine-containing HDACis.

Generally, the simplest way to introduce fluorine into the chemical structure of compounds is to use precursors that already contain the fluorine substituent. Derivatives synthesized by this approach mostly use basic coupling reactions such as alkylation, reductive amination, or an amide-coupling reaction. The advantage of these methods is that they are straightforward, easy to apply, and can thus also be performed in small research laboratories.

**Scheme 1:**
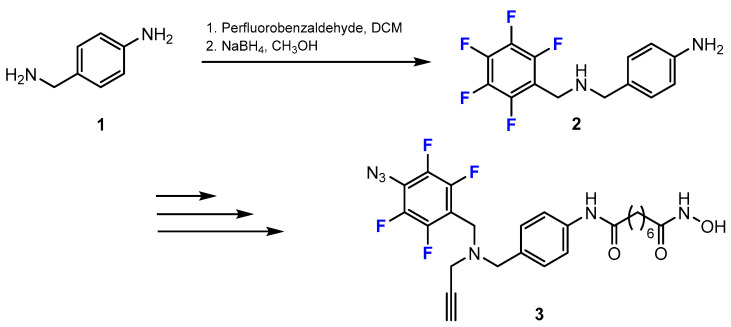
Fluorination in the synthetic pathway of fluorinated hydroxamic acids via reductive amination by Aboukhatwa et al. [33].

Aboukhatwa and co-workers [33] designed, synthesized, and evaluated the bioactivity of a series of novel chemically diverse photoreactive probes (PRPs). The general strategy for the synthesis of PRPs shows that the process of introducing fluoride-containing subunits into the designed compounds is by reductive amination. This approach included two steps (Scheme 1). The first was the reaction between the amine derivative and perfluorobenzaldehyde in dichloromethane (DCM). Then, the deprotonation of imines was carried out with sodium borohydride (NaBH_4_) in methanol (MeOH) to obtain the product containing a fluorine group.

**Scheme 2:**
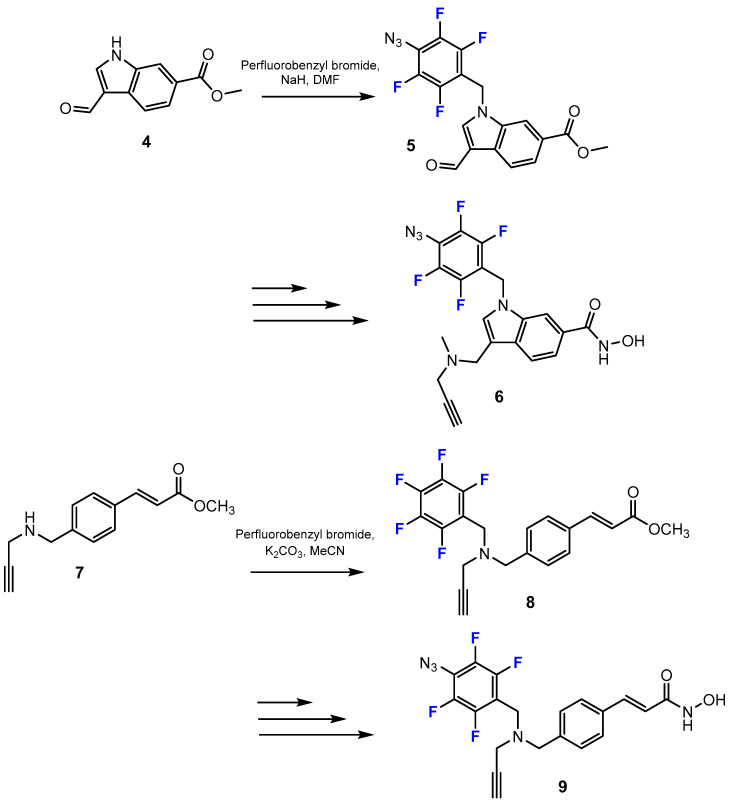
Fluorination in the synthetic pathway of fluorinated hydroxamic acids via alkylation by Aboukhatwa et al. [33].

Furthermore, in this study by Aboukhatwa [33], several novel indol-containing hydroxamic acids were synthesized using the *N*-alkylation of an indole moiety or secondary amine. The conditions of this step depended on the structure of the starting materials (Scheme 2). This reaction in the synthesis pathway of the final products bearing an indole ring was carried out in dimethylformamide (DMF) in the presence of sodium hydride (NaH). The process of introducing fluorine into a secondary amine, however, was performed under alkaline conditions (K_2_CO_3_), and acetonitrile (MeCN) proved to be an optimal solvent.

**Scheme 3:**
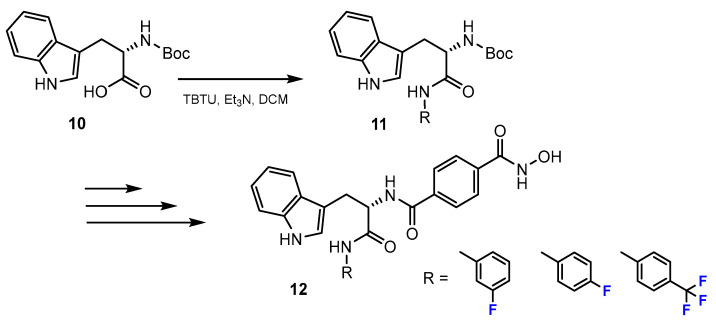
Fluorination in the synthetic pathway of fluorinated hydroxamic acids by Wang et al. [34].

In another study by Wang et al. [34], a series of hydroxamic acids bearing an indole ring were designed and synthesized. Fluorine-containing derivatives were obtained based on an amide-coupling reaction. This amide reaction was carried out in 2-(1*H*-benzotriazole-1-yl)-1,1,3,3-tetramethyluronium tetrafluoroborate (TBTU) and trimethylamine (Me_3_N) in DCM (Scheme 3).

**Scheme 4:**
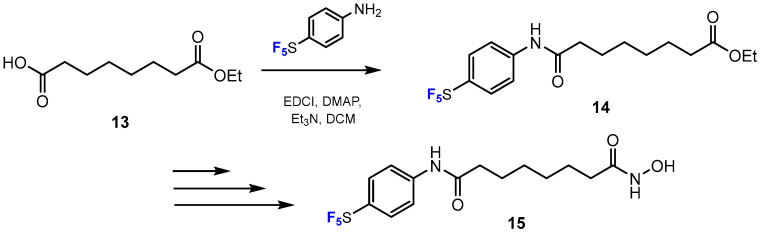
Fluorination in the synthetic pathway of fluorinated hydroxamic acids by Goehringer, Peng, et al. [35].

Using the same method, Goehringer, Peng, and co-authors [35] published a scheme for the synthesis of a novel pentafluorothio-substituted vorinostat-type HDACi (Scheme 4). This study described how the ethyl ester precursor SF_5_-SAHEt was obtained via amide-coupling reaction between ethyl hydrogen suberate and 4-pentafluorothioaniline in a moderate yield (63%). The catalysts used in this reaction were 1-ethyl-3-(3-dimethylaminopropyl)carbodiimide (EDCI), (dimethylamino)pyridine (DMAP), and triethylamine (Et_3_N). DCM was reported as a suitable solvent for this reaction.

**Scheme 5:**
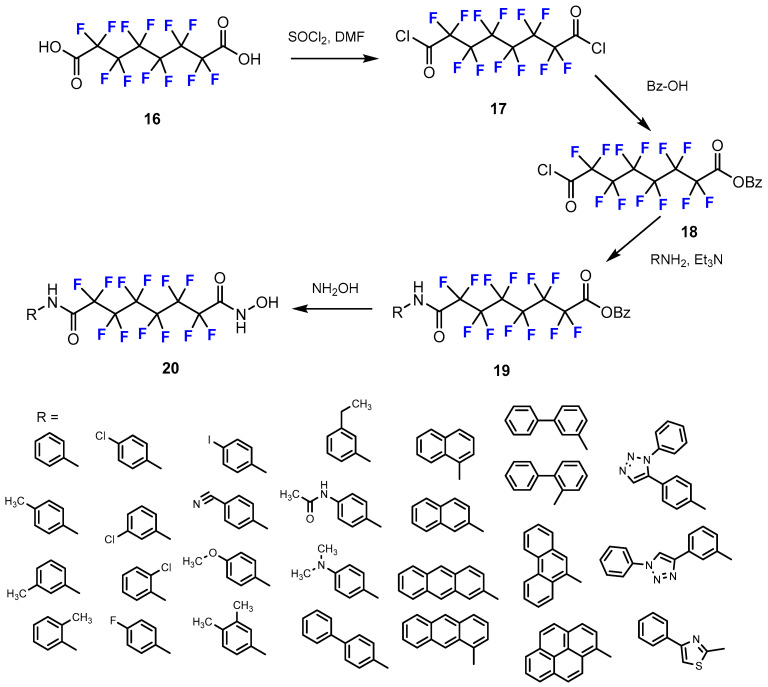
Fluorination in the synthetic pathway of fluorinated hydroxamic acids by Meyners et al. [36].

Meyners et al. [36] investigated a series of novel perfluorinated hydroxamic acids. These final compounds were synthesized by an amide-coupling reaction following a two-step procedure (Scheme 5). The first was the conversion from fluoro-containing carboxylic acids to the respective acyl chlorides using thionyl chloride (SOCl_2_). Secondly, the reaction between the acyl chlorides and the particular aromatic amines (RNH_2_) or heteroaromatic amines produced intermediate amides. The strategy pursued for the synthesis of these compounds was to use already fluorinated starting materials.

**Scheme 6:**
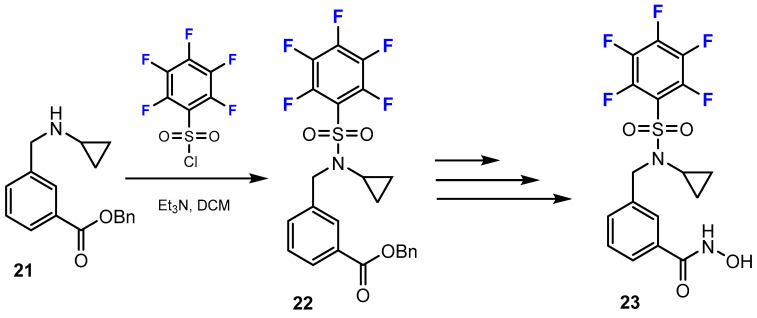
Fluorination in the synthetic pathway of fluorinated hydroxamic acids by Toutah et al. [37].

Toutah et al. [37] reported the synthetic pathways used to prepare hydroxamic acids containing fluorine substituents (Scheme 6). Fluorine was introduced into these compounds by amine sulfonylation employing polyhalogenated benzenesulfonyl chloride to yield sulfonamide precursors. This reaction was conducted smoothly in DCM and Et_3_N (yield: 61–91%).

**Scheme 7:**
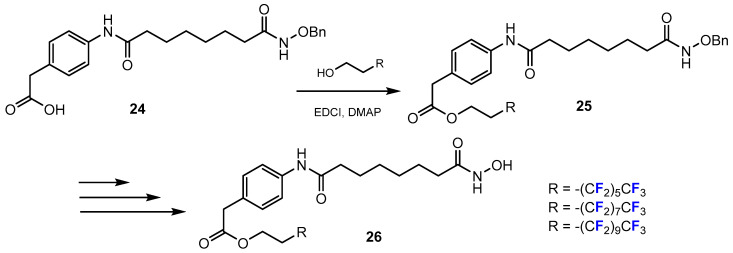
Fluorination in the synthetic pathway of fluorinated hydroxamic acids by Walton et al. [38].

A chemical reaction regularly used for the synthesis of fluorinated hydroxamic acids is represented by esterification. Walton et al. [38] reported several series of novel target compounds incorporating perfluorinated chains linked by a ester group. The ester bond plays an important role in connecting perfluorinated alkyl chains to a SAHA phenyl head group. Steglich esterification proceeded with the relevant alcohols in the presence of EDCI and DMAP, which produced intermediate compounds (Scheme 7).

**Scheme 8:**
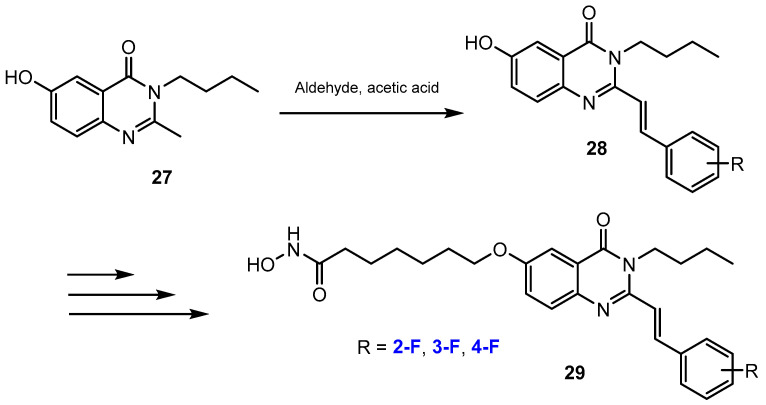
Fluorination in the synthetic pathway of fluorinated hydroxamic acids by Vu et al. [39].

Vu et al. [39] reported a synthetic pathway of several novel fluorinated *N*-hydroxyheptanamides containing quinazolin-4(3*H*)-ones. The fluorine substituent was introduced into these compounds by a nucleophilic substitution reaction between the 3-butyl-6-hydroxy-2-methylquinazolin-4(3*H*)-one and fluorine-containing aldehydes in acetic acid to afford intermediates in 61–82% yields (Scheme 8).

**Scheme 9:**
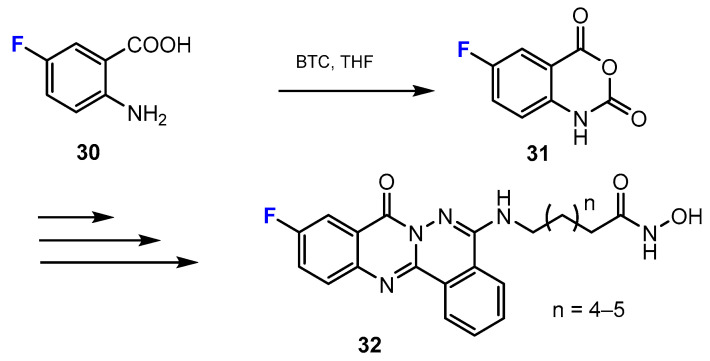
Fluorination in the synthetic pathway of fluorinated hydroxamic acids by Liu et al. [40].

Another strategy for the synthesis of novel fluorinated analogues covers the synthesis of heterocycles from the starting materials, which already contain the fluorine substituent. New phthalazino[1,2-*b*]-quinazolinone derivatives, which are multitarget HDAC inhibitors, were synthesized by Liu and co-authors [40]. The quinazolinone moieties were obtained from the reaction of 2-amino-5-fluorobenzoic acids and triphosgene (BTC, bis(trichloromethyl) carbonate). This reaction occurred smoothly in tetrahydrofurane (THF) (Scheme 9).

**Scheme 10:**
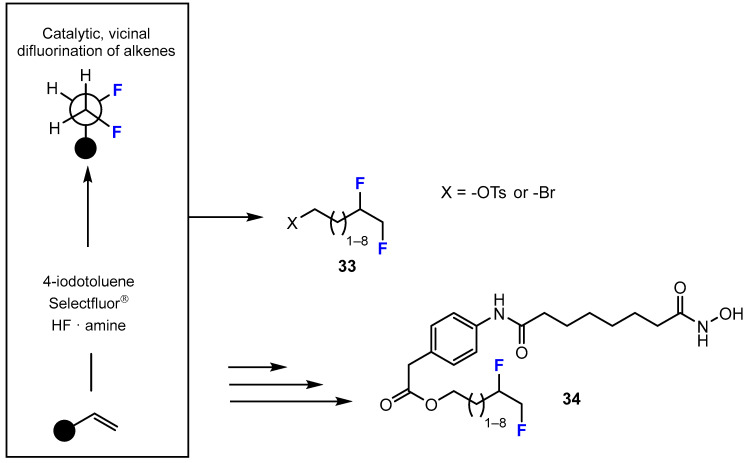
Fluorination in the synthetic pathway of fluorinated hydroxamic acids by Erdeljac et al. [41].

Fluorine-containing hydroxamic acids can also be synthesized by direct, catalytic vicinal difluorination. In the current research by Erdeljac et al. [41], novel fluorinated analogues of the HDACi vorinostat were synthesized from commercial sources or prepared from readily available starting materials (Scheme 10). The authors reported that 4-iodotoluene and Selectfluor^®^ were employed as an inexpensive organo catalyst and terminal oxidant, respectively. This reaction was carried in a solution mixture of amine:HF (1:5) in the presence of Et_3_N·3HF; Olah’s reagent, which served as fluoride source; and a Brønsted acid activator.

In more detail, a Teflon^®^ reaction vessel was loaded with the starting material, i.e., 4-iodotoluene, dichloroethan (DCE), an amine:HF (1:5) solution mixture of Olah’s reagent, Et_3_N·3HF, and Selectfluor^®^. The reaction vessel was sealed with a Teflon^®^ screw cap and the mixture was stirred at room temperature for 25 h. The yields of the intermediate products in this reaction were acceptable (39–94%).

**Scheme 11:**
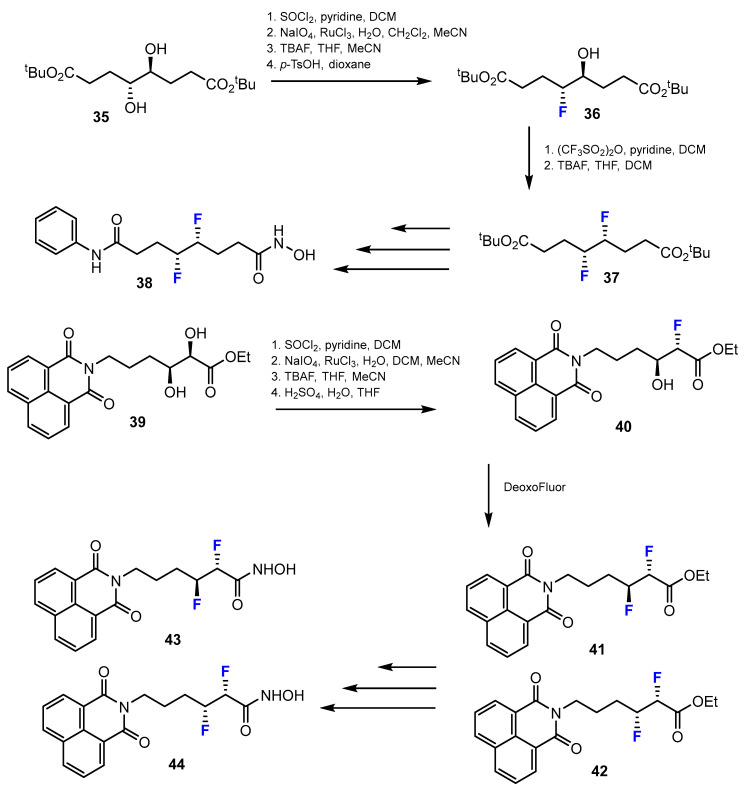
Fluorination in the synthetic pathway of fluorinated hydroxamic acids by Ariawan et al. [42].

A potential strategy for attaching fluorine atoms to the linker chain of difluorinated SAHA analogues was published by Ariawan et al. [42]. Several methods exist to introduce two fluorine atoms into hydroxamic acids (Scheme 11). The monofluorinated compounds were synthesized through a four-step sequence procedure via a cyclic sulfate intermediate. These four steps included: 1. SOCl_2_, pyridine, DCM; 2. NaIO_4_, RuCl_3_, H_2_O, DCM, MeCN; 3. TBAF, THF, MeCN; 4. *p*-TsOH, dioxane. The second hydroxyl group was activated as the triflate with (CF_3_SO_2_)_2_O, pyridine in DCM, which, when displaced with fluoride produced the difluorinated product in a moderate yield. This reaction was conducted in TBAF, THF, and DCM. Nevertheless, a naphthalimide-containing compound was achieved through chlorination (SOCl_2_, pyridine, DCM), cyclic sulfate formation (1. NaIO_4_, RuCl_3_, H_2_O, DCM, MeCN; 2. H_2_SO_4_, H_2_O, THF), followed by ring-opening with TBAF in THF and MeCN, to obtain the fluorohydrin. After that, the fluorohydrin was treated with DeoxoFluor to yield the difluorinated products.

One of the main goals to fluorinate the derivatives of known HDACis is to study their pharmacokinetic properties (see below). This is often accomplished by labeling with radioactive isotopes of fluorine such as ^18^F.

**Scheme 12:**
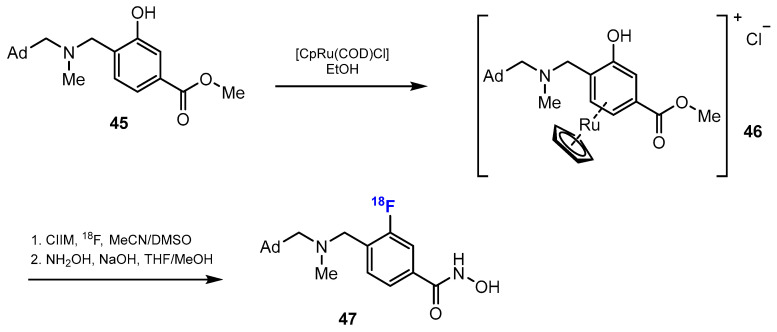
Fluorination in the synthetic pathway of fluorinated hydroxamic acids by Strebl et al. [43].

Therefore, Strebl et al. [43] reported the radiochemical synthesis of [^18^F]Bavarostat. While previous studies showed that the radiolabeling of hydroxamic acids such as Bavarostat were difficult to achieve because of protracted, inefficient, low-yielding methods, the authors followed a novel approach by using ruthenium-mediated radiofluorination. This pathway has various advantages, including high efficiency, yield, and specific radioactivity. Bavarostat can be radiolabeled with ^18^F by deoxyfluorination through the in situ formation of an air-stable ruthenium π-complex of the intermediate phenol precursor (Scheme 12). The labeling proceeding had high conversion (more than 70% radiochemical yield, as estimated by TLC). After HPLC (high-performance liquid chromatography) purification, the overall non-decay corrected radiochemical yield of [^18^F]Bavarostat was 8.1% (*n* = 2).

**Scheme 13:**
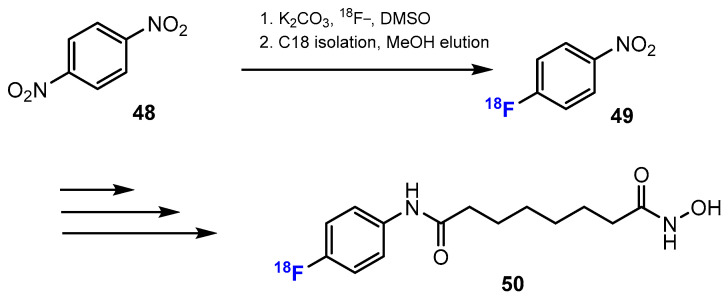
Fluorination in the synthetic pathway of fluorinated hydroxamic acids by Hendricks, Keliher, et al. [44].

In the study of Hendricks, Keliher, et al. [44] a synthetic strategy towards ^18^F-suberoylanilide hydroxamic acid (^18^F-SAHA) was developed. ^18^F (without carrier) was transferred to the starting material by the direct fluorination of 1,4-dinitrobenzene under optimized conditions for radiochemical synthesis and microwave heating at 120 ℃, in order to give ^18^F-labeled 1-fluoro-4-nitrobenzene (Scheme 13).

**Scheme 14:**
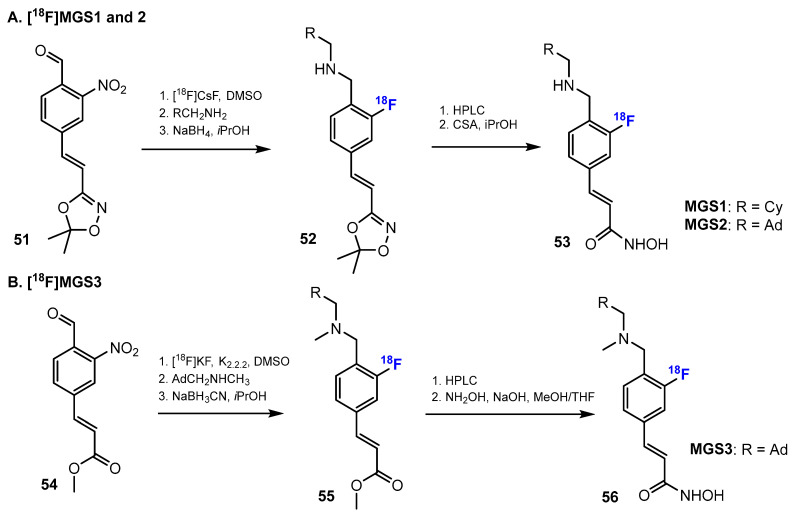
Fluorination in the two synthetic pathways (**A**,**B**) of fluorinated hydroxamic acids by Strebl et al. [45]. Radiochemical procedures for MGS1–2 (**A**) and MGS3 (**B**).

Recently, Strebl et al. [45] reported three novel fluorine-18 analogues of [^11^C]Martinostat ([^18^F]MGS1–3) via fluorination of the aromatic ring. The radiosynthesis of these radiotracers is illustrated in Scheme 14. In the synthetic routes of [^18^F]MGS1 and [^18^F]MGS2, the nitro group was replaced by ^18^F by using [^18^F]CsF, and DMSO was found as a suitable solvent. Meanwhile, in the synthetic pathway of [^18^F]MGS3, this reaction was carried out in DMSO with the presence of [^18^F]KF.

### 2.2. Benzamides

Benzamide-containing HDACis are the second most studied class of derivatives along with hydroxamic acids. Inhibitors with 2-aminobenzamide as ZBG show potential activity against HDAC class I, such as HDAC3 and HDAC8 [46]. There are several methods for the synthesis of fluorinated benzamides. The most common way to introduce fluorine into the intended compounds is again to use starting materials that are already fluorinated. In this context, simple reactions such as amide-coupling are used.

**Scheme 15:**
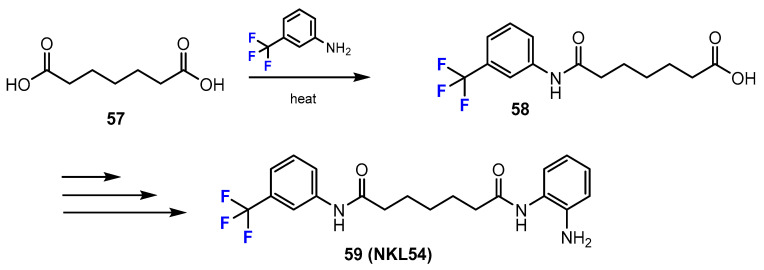
Fluorination in the synthetic pathway of fluorinated benzamides by Jayathilaka et al. [47].

Jayathilaka and co-workers [47] investigated the synthetic pathway to give the fluorinated analogue NKL54 (Scheme 15). The trifluoromethyl group was introduced into the designed compounds using fluorinated aniline. The amide product is generally formed from the carboxylic acid and the aromatic amine in a 68% yield.

**Scheme 16:**
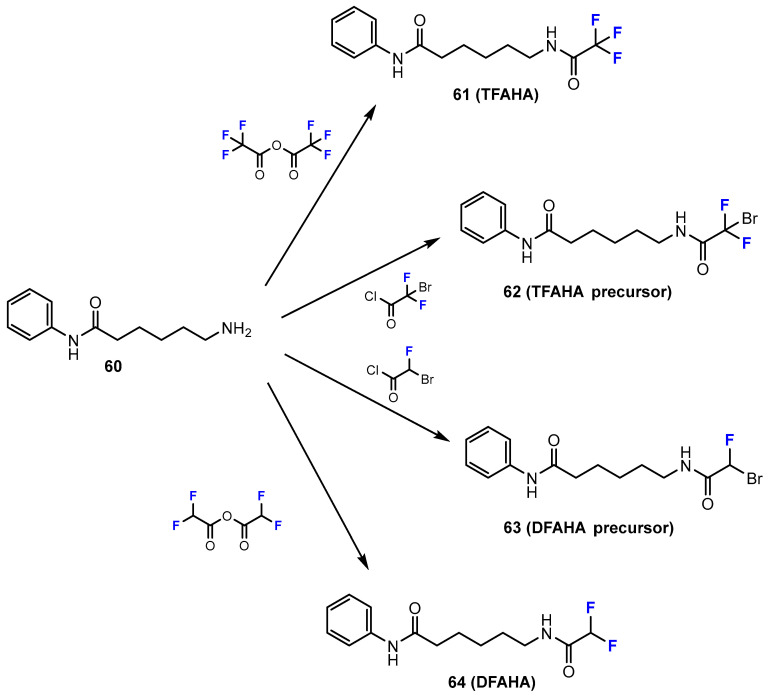
Fluorination in the synthetic pathway of fluorinated benzamides by Bonomi et al. [48].

In the research of Bonomi et al. [48], the synthesis of novel histone deacetylase radio-tracers for positron-emission tomography (PET) imaging, including DFAHA and TFAHA precursors, was reported. The amide bonds of DFAHA and TFAHA were formed using particular anhydrides in DCM at room temperature (Scheme 16). The TFAHA precursor was synthesized in pyridine and acetyl chloride, while the reaction of the amine with bromofluoroacetyl chloride and triethylamine in DCM obtained the DFAHA precursor in a modest yield (15%).

**Scheme 17:**
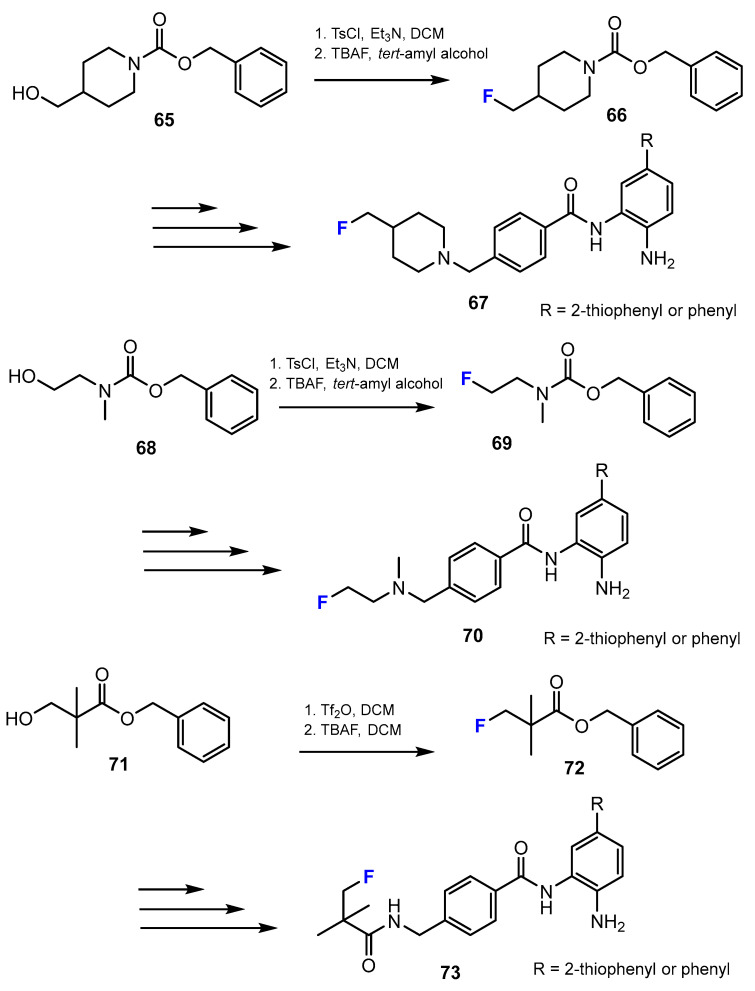
Fluorination in the synthetic pathway of fluorinated benzamides by La et al. [49].

In the current research of La and co-authors [49], the synthesis of novel fluorinated benzamide compounds as potential HDACis was described. The final products with the cap containing a fluorine moiety were obtained via substitution of the hydroxyl functional group to a fluorine substituent applying tosylate compounds (Scheme 17). DCM and *tert*-amyl alcohol were found as suitable solvents for this reaction.

**Scheme 18:**
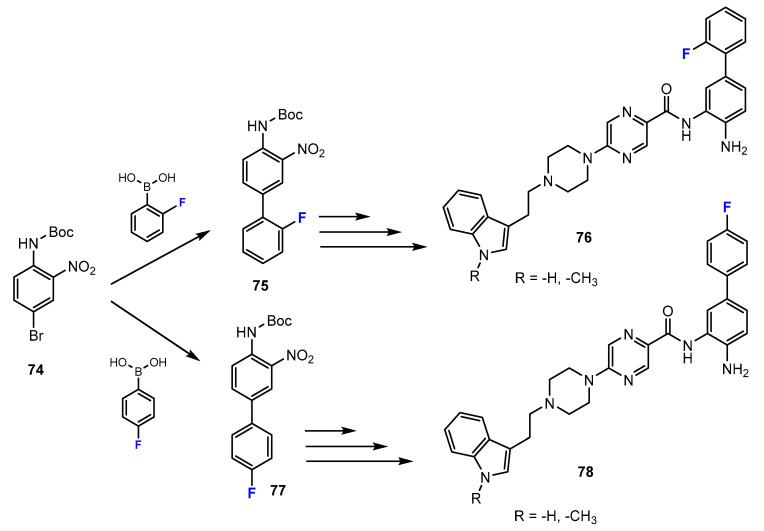
Fluorination in the synthetic pathway of fluorinated benzamides via Suzuki reaction by Ibrahim et al. [50].

In the study by Ibrahim et al. [50], a series of fluorine-containing compounds was prepared through Suzuki coupling reaction between the Boc-protected 4-bromo-2-nitroaniline and the corresponding fluorinated boronic acid compounds (Scheme 18). Tetrakis P(Ph)_3_Pd served as an efficient catalyst, and 1,2-dimethoxyethane was reported to be the optimal solvent. Sodium carbonate was added to the reaction mixture, which was needed for the sequestration of acid generated.

**Scheme 19:**
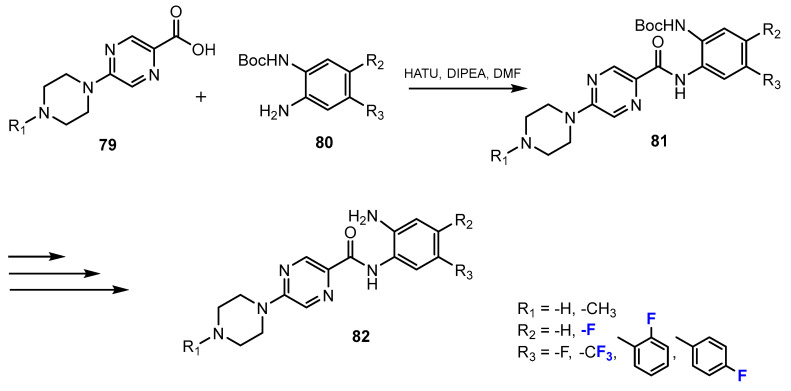
Fluorination in the synthetic pathway of fluorinated benzamides via amide-coupling reactions by Ibrahim et al. [50].

Also in this research [50], the authors reported another method for the synthesis of fluorinated 2-aminobenzamides using amide coupling. The amide intermediates were prepared by the reactions between the carboxylic acid derivatives and 1,2-phenylenediamines employing the coupling reagent HATU (hexafluorophosphate azabenzotriazole tetramethyl uronium) and *N*,*N*-diisopropylethylamine (DIPEA) as the base (Scheme 19). DMF was described to be the optimal solvent for these amide-forming reactions.

**Scheme 20:**
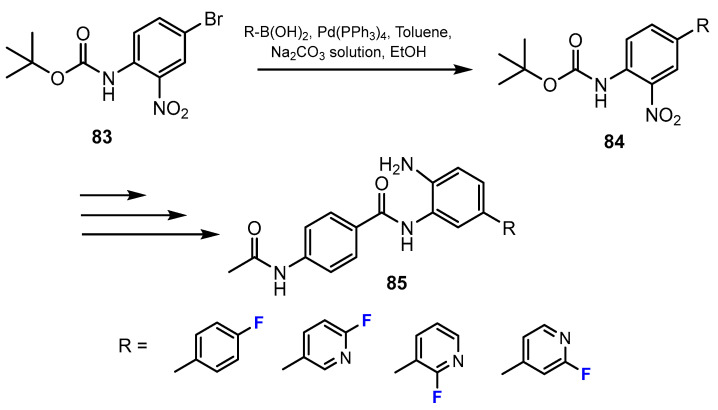
Fluorination in the synthetic pathway of fluorinated benzamides by Schäker-Hübner et al. [51].

Schäker-Hübner and co-workers [51] also synthesized a series of novel benzamides. The Boc-protected intermediates containing fluorine were obtained via the Suzuki reaction of *tert*-butyl-(4-bromo-2-nitrophenyl)carbamate with alkyl boronic acids (Scheme 20). Tetrakis(triphenylphosphine)palladium(0) (Pd(PPh_3_)_4_) was reported as a satisfactory catalyst for these coupling reactions. The mixture of toluene and ethanol was adduced as solvent.

**Scheme 21:**
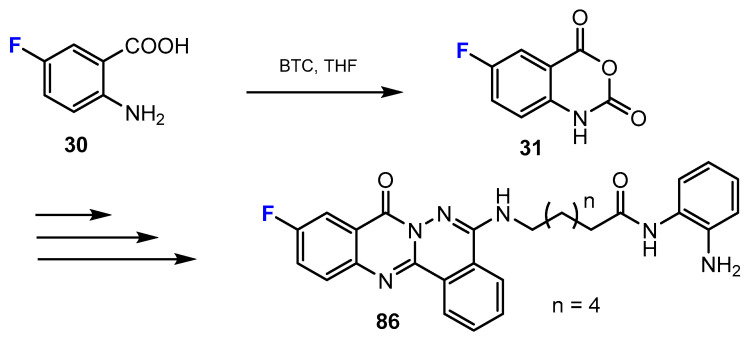
Fluorination in the synthetic pathway of fluorinated benzamides by Liu et al. [40].

In the research work of Liu and co-workers [40], as mentioned above among the hydroxamic acid derivatives (Scheme 9), the authors used the same strategy as for the synthesis of fluorinated hydroxamic acids to obtain new fluorinated benzamides. The quinazolinone ring was synthesized from the reaction between the initial 2-aminobenzoic acids with a fluorine substituent and triphosgene (Scheme 21).

**Scheme 22:**
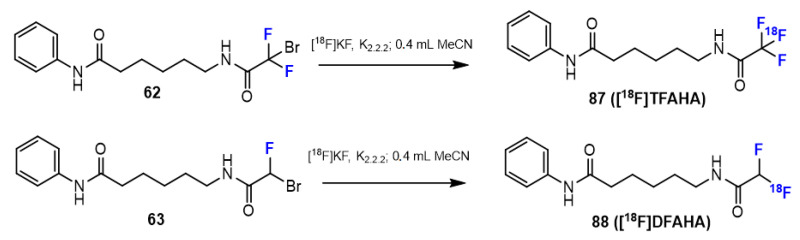
Fluorination in the radiosynthetic pathway of fluorinated benzamides by Bonomi et al. [48].

Similar to hydroxamic acids (see above), some studies have also been performed to synthesize fluorinated benzamide derivatives containing the radiolabel ^18^F. Moreover, in the study of Bonomi et al. [48], the authors investigated a series of novel histone deacetylase radiotracers for PET imaging, including [^18^F]DFAHA and [^18^F]TFAHA (Scheme 22). The target benzamides were synthesized in radiochemical yields of 25% and 22%, respectively. The chemical structures of the radioactive products were confirmed by nuclear magnetic resonance (NMR) spectroscopy, and the purity was more than 95%, which was determined by analytical radio-HPLC.

**Scheme 23:**
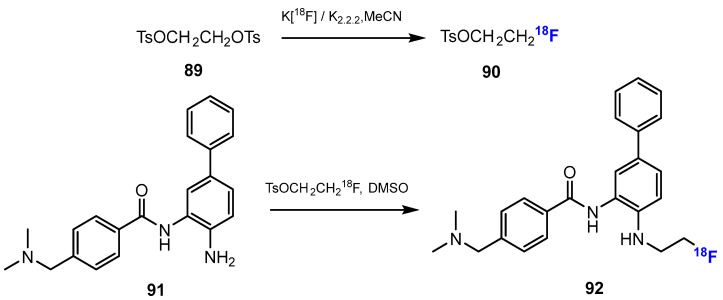
Fluorination in the radiosynthetic pathway of [^18^F] Fluoroethyl-INER1577 by Li et al. [52].

Li and co-authors [52] investigated the synthesis of [^18^F] Fluoroethyl-INER1577 (i.e., INER1577-3, **92**, Scheme 23). The first step was the fluorination of ethane-1,2-diyl bis(4-methylbenzenesulfonate) (TsOCH_2_CH_2_OTs) with K[^18^F] and kryptofix 2.2.2 (K2.2.2). This reaction was conducted smoothly in MeCN at 90 °C for 10 min. The radiochemical yield of TsOCH_2_CH_2_O^18^F was approximately 70%. Then, the intermediate reacted with INER1577 (**91**) in DMSO at 100 °C to produce [^18^F]Fluoroethyl-INER1577 (**92**). After 60 min, the radiochemical yield of [^18^F]Fluoroethyl-INER1577 was approximately 5–10%, and both chemical and radiochemical purities were more than 99%.

**Scheme 24:**
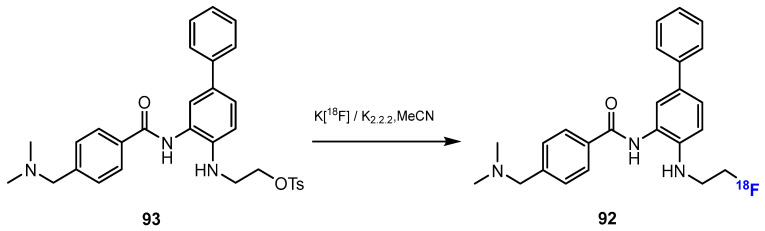
Fluorination in the radiosynthetic pathway of [^18^F] Fluoroethyl-INER1577 by Li et al. [53].

In another work by Li et al. [53], [^18^F] INER1577-3 was synthesized by the one-step method (Scheme 24). This reaction was carried out with [^18^F]fluoride without carrier addition in the presence of K_2_CO_3_ and K2.2.2 in 85% MeCN/water. The optimum temperature of this step was approximately 100 °C. The radiochemical purity of the final product was more than 95% (determined by the analytical RP-HPLC method).

### 2.3. Thiols

In recent years, besides the research on HDACis based on hydroxamic acids or benzamides, thiol derivatives have also been attracting much attention. This may be related to the ability of thiols to chelate zinc well [54]. Several research studies on fluorinated thiol derivatives have also been published.

**Scheme 25:**
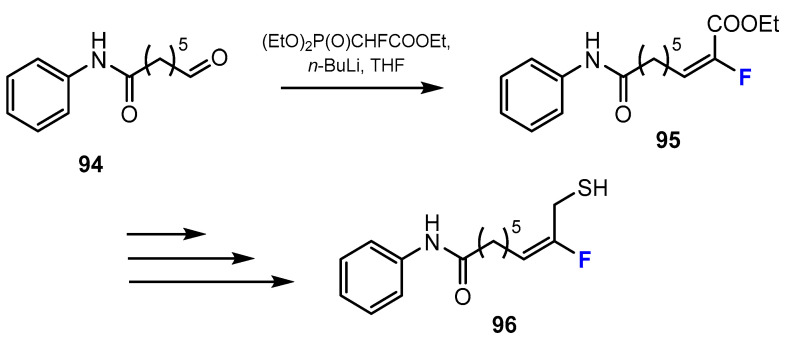
Fluorination in the synthetic pathway of fluorinated thiols by Chuman et al. [55].

For example, Chuman et al. [55] described methods for the synthesis of fluoroalkene-containing HDACis. As outlined in Scheme 25, the designed thiols were obtained through the reactions between the intermediate aldehyde and triethyl 2-fluoro-2-phosphonoacetate (EtO)_2_P(O)CHFCOOEt to obtain fluoroalkene in the presence of *n*-butyllithium (*n*-BuLi) in THF. Interestingly, the advantage of this method is that the fluoroalkene was formed with *E*-selectivity.

**Scheme 26:**
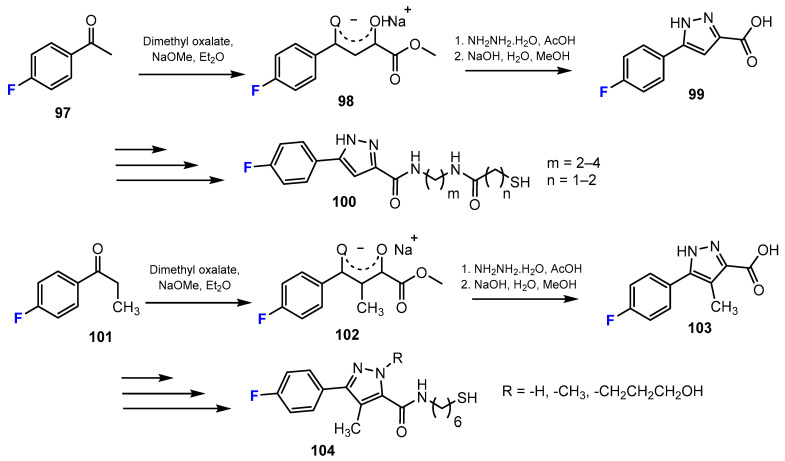
Fluorination in the synthetic pathway of fluorinated thiols by Wen et al. [56].

In another study of Wen et al. [56], a series of novel thiol-based HDACis was synthesized. Fluorine was introduced into the designed compounds by condensation reactions. These reactions occurred between 4-fluoroacetophenone or 4-fluoropropiophenone and dimethyl oxalate (Scheme 26). The intermediates were transferred to phenylpyrazole esters via the treatment of these compounds with hydrazine hydrate in acetic acid, followed by hydrolysis in the presence of sodium hydroxide (NaOH) in MeOH, to produce carboxylic acids.

### 2.4. Short-Chain Fatty Acids

Several natural short-chain fatty acids have shown promise to serve as potential HDACis [57,58]. There were also studies on fluorine-containing fatty acids comparing the HDAC inhibitory activities of natural amino acids and fluorinated amino acid derivatives.

**Scheme 27:**
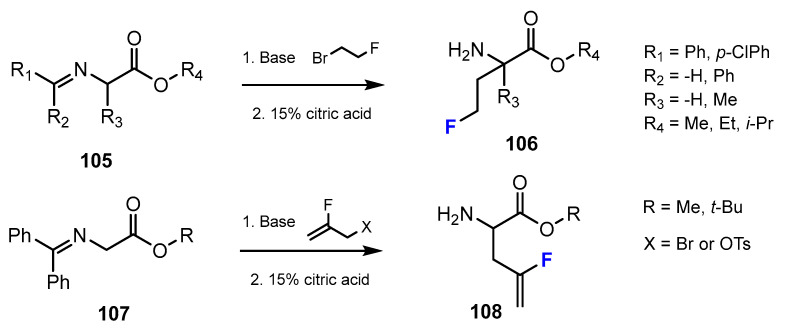
Fluorination in the synthetic pathway of fluorinated short-chain fatty acids via alkylation by Lübke et al. [59].

A series of fluorinated amino acid esters were reported in the research study of Lübke et al. [59]. These potent and selective HDACis were synthesized from several unnatural amino acids bearing monofluoroalkyl side chains (Scheme 27). The process of introducing fluorine-containing moieties into the designed compounds is accomplished through alkylation.

**Scheme 28:**
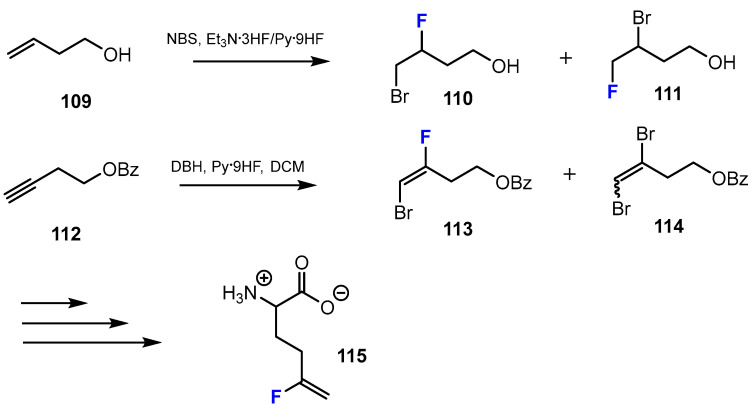
Fluorination in the synthetic pathway of fluorinated short-chain fatty acids via bromofluorination by Lübke et al. [59].

A second method described by Lübke et al. [59] to introduce fluorine into the chemical structure of amino acid esters was to use an addition reaction (Scheme 28). The fluorinated intermediates were achieved by bromofluorination with 1,3-dibromohydantoine (DBH) and Olah’s reagent (Py·9HF) or *N*-bromosuccinimide (NBS), Et_3_N·3HF. After this step, a mixture of bromofluoro ester and dibromides was obtained. Following separation gave the desired products.

The authors reported that fluorine substituents played an important role in the interaction of HDACis with the active site of the enzymes. This research study also showed that a fluoro vinyl moiety had a similar role to an apolar amide group in terms of physical or chemical properties, which can lead to similar biological properties [59].

### 2.5. Cyclic Peptides

**Figure 2:**
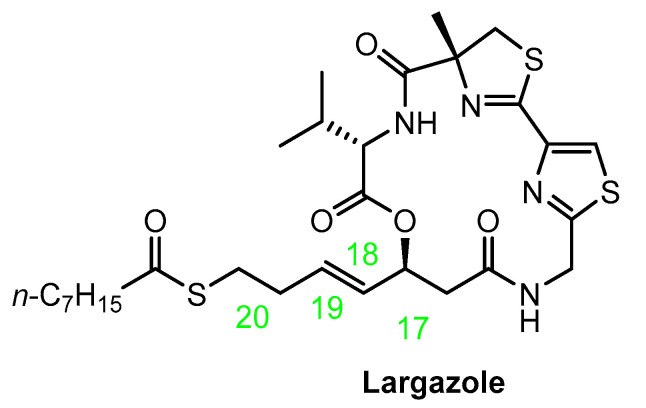
Chemical structure of largazole.

There are also fluorinated cyclic peptides with potential activity as HDACis. Such derivatives were recently designed and synthesized by Zhang, Liu, Gao, and co-workers [60,61]. All compounds reported in these studies were analogues of largazole (Figure 2). This is a cyclic depsipeptide derived from a cyanobacterium of the genus *Symploca*. It effectively inhibits the HDACs of class I [62].

**Scheme 29:**
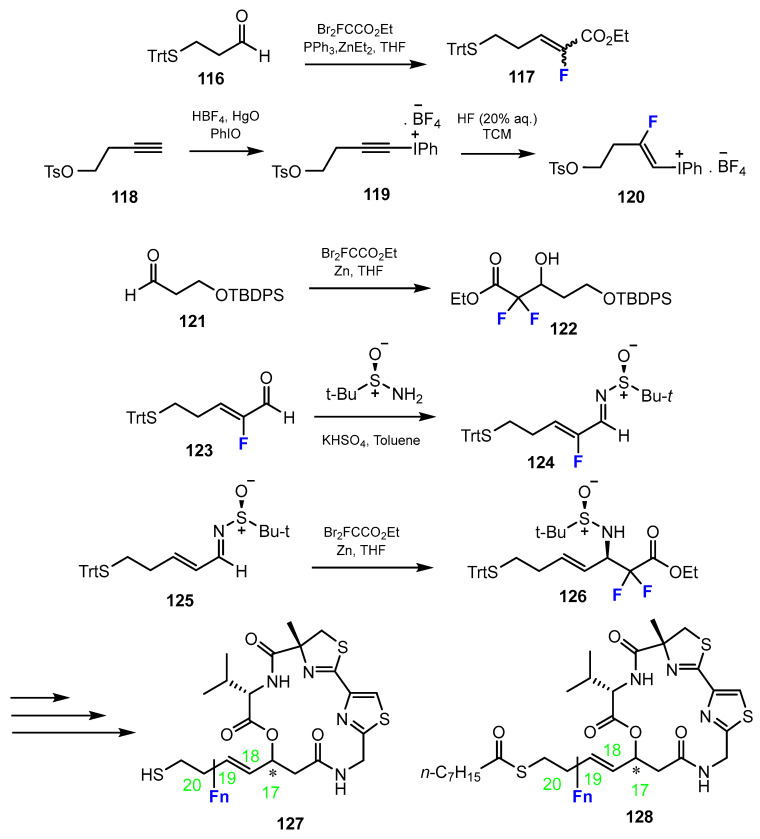
Fluorination in the synthetic pathway of fluorinated cyclic peptides by Zhang, Liu, Gao, et al. [60,61].

The fluorine-containing side chain fragments were provided via several types of reactions, which are all described in Scheme 29 below. The first step to introduce the fluorine for the design of cyclic peptides was the alkylation with ethyl 2-bromo-2,2-difluoroacetate in the presence of triphenylphosphine and diethylzinc in THF. An alternative method for fluorination was through a Reformatsky reaction with ethyl 2-bromo-2,2-difluoroacetate, zinc in THF. Another pathway involved a two-step process. The first step was the preparation of 1-alkynyl(phenyl)iodonium tetrafluoroborate from alkyne. Then, the intermediate was fluorinated by the reaction with 20% HF aqueous solution in CHCl_3_. Moreover, the use of starting materials that already contain the fluorine substituent is also feasible.

## 3. Biological Significance and Purpose of Fluorination in HDACis

### 3.1. Potency

The potency of a drug is often a very important parameter in the medicinal chemistry design of novel compounds [63]. Potency generally refers to the effectivity of an active ingredient. This is expressed by the concentration required to achieve a certain pharmacological effect, e.g., enzyme inhibition or cytotoxicity [64]. Increasing the potency can ensure both engagement with the target and the avoidance of off-target activity. If it is possible to use a drug at a lower dose due to its high potency, this may be associated with less toxicity. The particular target plays a role in each case, in order to prove which transformations of the chemical structure ultimately also lead to an increase in potency. Nevertheless, some modifications occur with high frequency in different drug classes and contribute to an improvement in potency [65]. These structural alterations include, among others, fluorination. Recent examples where fluorination has had an impact on the efficacy of HDACis are described below.

Erdeljac and co-workers [41] investigated the influence of the introduction of the chiral 1,2-difluoroethylene unit as a hybrid bioisostere of the trifluoromethyl and ethyl (BITE) group in a series of eight vorinostat derivatives (Scheme 10). Compared to pure vorinostat, the latter additionally possess an ester moiety with different alkyl lengths of the alcohol component. The potency of the fluorine-containing BITE compounds with respect to the in vitro inhibition of HDAC1 and HDAC6 was compared with that of the non-fluorinated ethyl congeners.

Against HDAC1, all eight fluorinated homologs were more potent than the corresponding ethyl derivatives. Four of the fluorinated compounds (*n* = 1–4) even exceeded the potency of the clinically approved vorinostat. Targeting the isoenzyme HDAC6, six of the 1,2-difluoroethylene-bearing compounds were more active than the respective ethyl derivatives and vorinostat. However, the potency against HDAC1 and HDAC6 generally decreased with the elongation of the methylene spacer in both series.

In a related investigation of the trifluoromethyl group as a bioisostere to the ethyl substituent, the authors recognized a low lipophilicity in this particular compound as a result of fluorination. Furthermore, a relationship between lipophilicity and passive permeability was deduced. The introduction of fluorine also had an effect on the solubility of the compounds. However, no consistent correlation with an increasing degree of fluorination could be established, but a heterogeneous order [66].

Based on these results, the authors judged the BITE group to be a promising bioisostere for the development of potent HDACis. Moreover, they suggested that the concept of 1,2-difluoroethylene derivatives can be successfully applied to other small molecules in the future [41].

A similar approach was already taken by Walton et al. [38] in designing a series of six hydroxamic acids derived from vorinostat with perfluorinated alkyl side chains of different lengths (Scheme 7). The antiproliferative effect of the compounds was investigated against ovarian cancer cells A2780. Five of the six perfluorinated compounds showed cytotoxicity in the single-digit micromolar range, two of which were minimally better and another two of which were significantly more potent than vorinostat. This proves a positive effect of the introduction of perfluorinated alkyl side chains. In this context, the authors suggested that compounds with a longer perfluorinated chain have lower uptake in cancer cells due to their decreased solubility. This in turn leads to less toxicity.

Also in the work of Goehringer, Peng, and colleagues [35], the structure of the clinically approved drug vorinostat was altered. In particular, the modification is based on the introduction of a 4-pentafluorosulfanyl substituent leading to a vorinostat derivative with a para-SF_5_ aryl cap (Scheme 4). The authors justified their decision for the SF_5_-group with the fact that it is a stable imitator of negatively charged biomolecules, due to its lipophilicity and electron-withdrawing properties [67]. The growth inhibitory activity of SF_5_-vorinostat against six human cell lines was compared with that of vorinostat and revealed a beneficial effect on increasing potency.

While SF_5_-vorinostat exhibited approximately the same cytotoxicity as the parent compound against the human prostate cell line DU145, it had approximately two-fold higher potency in the other cell lines tested (hepatocellular carcinoma Hep-G2, and T-cell leukemia/lymphoma cell lines Jurkat, Hut78, SupT11, and SMZ1) due to an IC_50_ value half as high. In all six cell lines, the IC_50_ value of SF_5_-vorinostat was in the low single-digit micromolar range.

**Figure 3:**
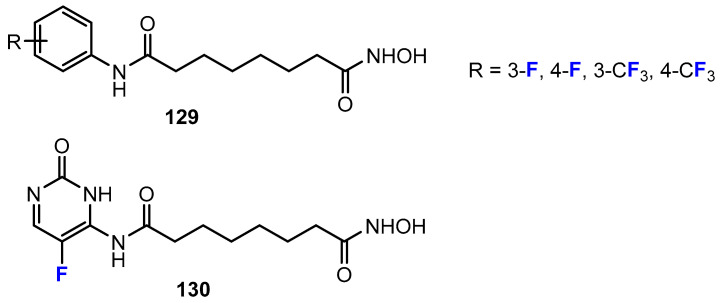
Chemical structures of fluorinated hydroxamic acids by Salmi-Smail et al. [68].

Similar effects were detected for the respective trifluoromethyl analogue (Figure 3) in the study by Salmi-Smail et al. [68]. This showed approximately equal antiproliferative activity as vorinostat in the breast carcinoma SKBR3, chronic myelogenous leukemia K562, and acute myelogenous leukemia HL60 cell lines. More importantly, CF_3_-vorinostat was superior by a factor of approximately 1.4–1.8 in colon carcinoma HT29, histiocytic lymphoma U937, and Jurkat JA16 cell lines. The 4F-vorinostat derivative (Figure 3) was only tested in the cell lines SKBR3, HT29, and U937. Its IC_50_ value was not better than that of vorinostat; however, it was still in the low single-digit micromolar range. In contrast, 3F-vorinostat (Figure 3) was equivalent or slightly superior to vorinostat in these three cell lines.

Zhang, Liu, Gao et al. [60,61] adduced the HDACi largazole (Figure 2) and performed a fluoroscan to extend the structural diversity. This resulted in largazole thiol derivatives with different fluorination in the thiolate side chain (Scheme 29). Derivatives of **116** exhibiting single fluorination at positions 18 and 19, respectively, or the compound which was double fluorinated at position 20, were investigated. The efficacy of the compounds to inhibit human HDACs 1–3, 6, 8 was investigated. In comparison with largazole, these three fluorinated derivatives were more potent. It should be mentioned that largazole thiol exerted stronger effects than largazole (Figure 2) itself. However, the derivative fluorosubstituted in the C19 position even exceeded its activity.

**Figure 4:**
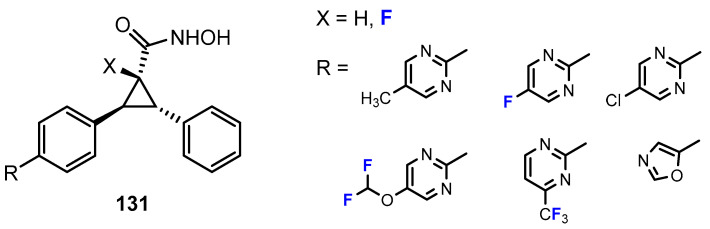
Chemical structures of fluorinated hydroxamic acids by Luckhurst [69].

The research of Luckhurst and co-workers [69] was focused on the development of tetrasubstituted cyclopropane hydroxamic acids as HDACis (Figure 4). Formally starting with the trisubstituted compounds, they took the approach of introducing a fluorine substituent leading to the tetrasubstituted congeners. This was based on the assumption that the fluorine at the Cα adjacent to the hydroxamic acid would lead to enhanced acidity, and thus, increase activity. The authors reported on six pairs of compounds, each with and without the fluorine substituent. The potency of these compounds to inhibit the HDAC1 enzyme was investigated. The working hypothesis was confirmed, as all fluorinated derivatives were two to nine times more active than the unfluorinated analogues. In the series of compounds, the pyrimidine capping group was also modified. In this context, fluorination in the form of the introduction of a single fluorine substituent, a difluoromethoxy or a trifluoromethyl group, was considered. However, only minimal effects on activity were observed in this regard.

**Figure 5:**
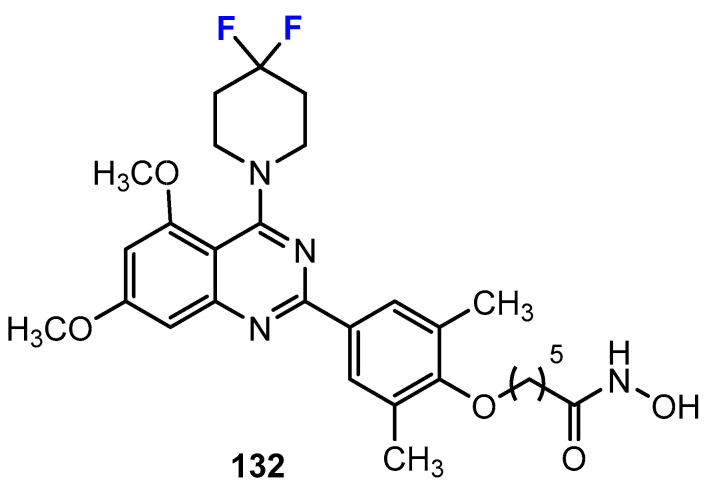
Chemical structure of fluorinated hydroxamic acid by Yao, Li et al. [70].

The study by Yao, Li, and coworkers [70] reported on HDACis, in which structural modifications were made to the surface recognition cap, including the introduction of a 4,4-difluoropiperidin-1-yl residue (Figure 5). However, the fluorinated compounds only showed moderate antiproliferative activity against MDA-MB-231 cells.

**Figure 6:**
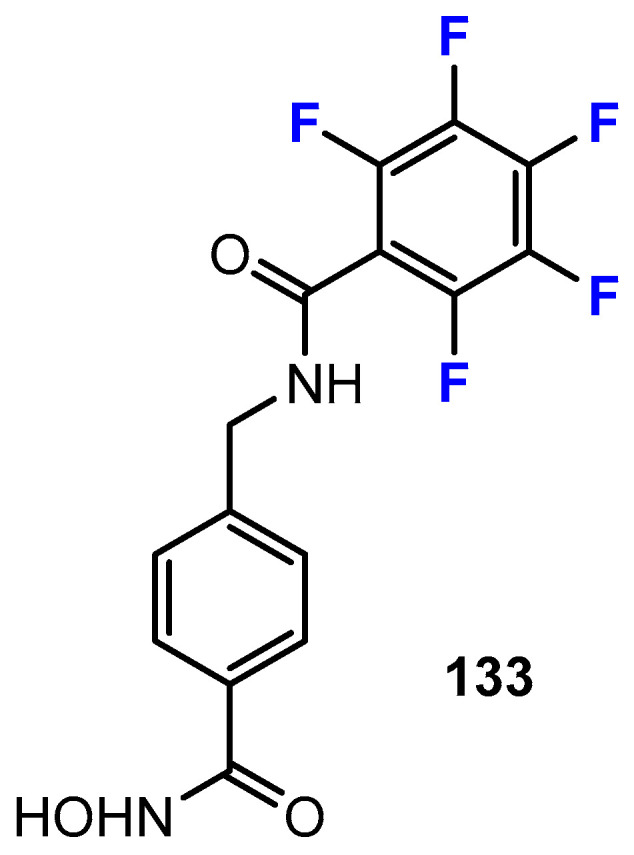
Chemical structure of fluorinated hydroxamic acid (PTG-0861) by Gawel, Shouksmith, Raouf, Nawar et al. [71].

Gawel, Shouksmith, Raouf, Nawar, and co-workers [71] described some HDACis belonging to hydroxamic acids with different fluorination patterns in the phenyl cap group. The cap moiety serves to anchor the inhibitor to the outside of the active center of the HDAC. In their work, the authors also introduced derivatives with a pentafluorophenyl group (Figure 6). The cytotoxicity was investigated using the leukemia cell line MV4-11.

The *p*-fluorinated compound showed cytotoxicity with an IC_50_ value of 3.64 μM. Interestingly, the compound with a trifluoromethyl substituent in the para-position was even more potent (0.88 μM). The compound with a five-fold fluorination of the aromatic residue (pentafluorobenzene derivative, 1.51 μM) was also stronger than the monofluorinated derivative. This trend could indicate a positive effect on potency upon increasing fluorination. A comparison of the other pentafluoro compounds in their study (i.e., compounds **60** and **84** in the original work: 1.85 μM and 7.37 μM, respectively) with the corresponding *p*-tert-butyl-substituted compounds (i.e., compounds **68** and **83** in the original work: 0.57 μM and 8.57 μM, respectively) only partially confirms this. Here, the different spacers between the cap group and the hydroxamic acid moiety seem to have an influence. This can also be seen in the pentafluoro compounds (i.e., compounds **100** and **101** in the original work: 0.32 μM and 0.76 μM, respectively), which induced even stronger cytotoxicity against MV4-11 cells than citarinostat (1.24 μM).

**Figure 7:**
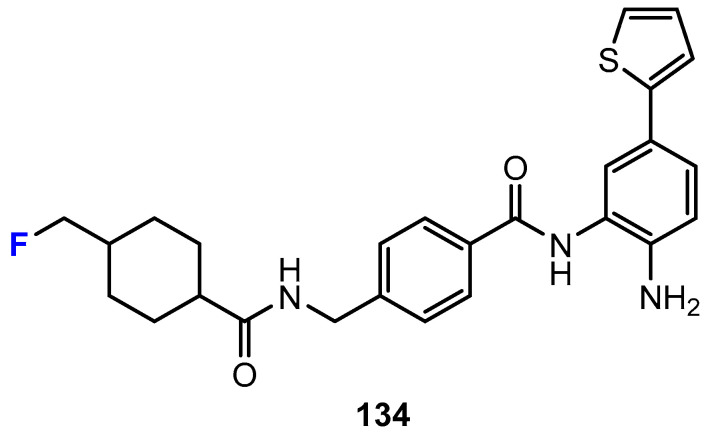
Chemical structure of fluorinated benzamide (CBUD-1001) by Kim, La et al. [72].

A HDACi also fluorinated in the cap group was described in the work of Kim, La and co-workers [72]—in particular, a fluorinated aminophenyl-benzamide-based drug (Figure 7). In addition, in the chemical structure of the HDACi, the zinc-binding group was substituted by a “foot pocket” unit, that is, a thiophen-2-yl substituent. The compound was designed to specifically inhibit HDAC1. It exhibited promising cytotoxicity against the colorectal cancer cell lines HCT116 (IC_50_ = 5.59 μM) and DLD-1 (IC_50_ = 4.05 μM). Remarkably, the activity against normal intestinal epithelial cells (ICEs, IC_50_ = 18.38 μM) was approximately four times less pronounced, demonstrating some kind of selectivity towards colon cancer cells. Based on these results, the authors concluded that the biarylbenzamide structure, as well as fluorination within the cap moiety, represent a promising strategy for the development of potent HDAC inhibitors.

Schäker-Hübner et al. [51] also studied the effect of fluorination of the “foot pocket” unit attached to the zinc-binding domain (Scheme 20). They observed ambivalent results due to fluorination when the inhibitory potential of the newly synthesized fluorine-containing compounds **85** was investigated. The introduction of a 4-fluorophenyl substituent resulted in a slightly more potent inhibition of the isoenzymes HDAC1-3 compared to the phenyl parent compound. However, when a fluorine substituent was additionally introduced at positions 2 or 6 starting from the pyridin-3-yl substituted compound, the inhibition of HDAC1-3 was lower. The authors investigated that fluorination on the aromatic system increases lipophilicity, and thus, decreases solubility, which could in turn explain a poorer inhibitory effect. For the 2-fluoropyridin-4-yl derivative, compared with the non-fluorinated pyridin-4-yl compound, inhibition was reduced towards HDAC1-2 and enhanced towards HDAC3. Depending on the position where the fluorine substituent is introduced, not only the potency can be increased, but also the selectivity can be fine-tuned.

### 3.2. Selectivity

In addition to potency, another parameter that plays an important role in any drug design is selectivity [73]. Selectivity generally describes the extent to which a drug binds to targets that are different than the desired target (e.g., enzymes, receptors, ion channels) or acts on different tissues (e.g., cancerous vs. non-cancerous). High selectivity means that few additional targets are bound. Ideally, binding occurs only to the target through which the pharmacological effect is exerted and not to other targets that may be associated with undesirable side effects.

In the development of HDACis as antitumor compounds, it is important that the compounds induce their effects on cancer cells and not on healthy cells. On the other hand, there are also efforts to create selectivity towards the different HDAC isoenzymes [74]. This is based on the fact that not all HDAC isoforms are overexpressed in all types of cancers [75].

For the vorinostat derivative **26** bearing perfluorinated alkyl side chains (Scheme 7), as reported in the study of Walton et al. [38], approximately five-fold selectivity over ovarian carcinoma cells A2780 can be seen for the compound with the polyfluorinated C10 chain compared to non-cancerous HEK cells. Since for the unfluorinated analogue the selectivity was approximately two and a half times, fluorination could lead to the doubling of the selectivity. With the parent compound vorinostat, there was almost no selectivity for the observed tumor cells. For the sake of completeness, however, it must be mentioned that for the other derivatives with C8 and C12 chains, the selectivity with respect to tumor cells could not be increased by fluorination. Ultimately, selectivity towards tumor cells can be achieved if there is selectivity with respect to tumor-specific targets [76]. In the development of HDACis, it takes an important role to specifically address certain isoforms of HDAC with the newly developed compounds.

**Figure 8:**
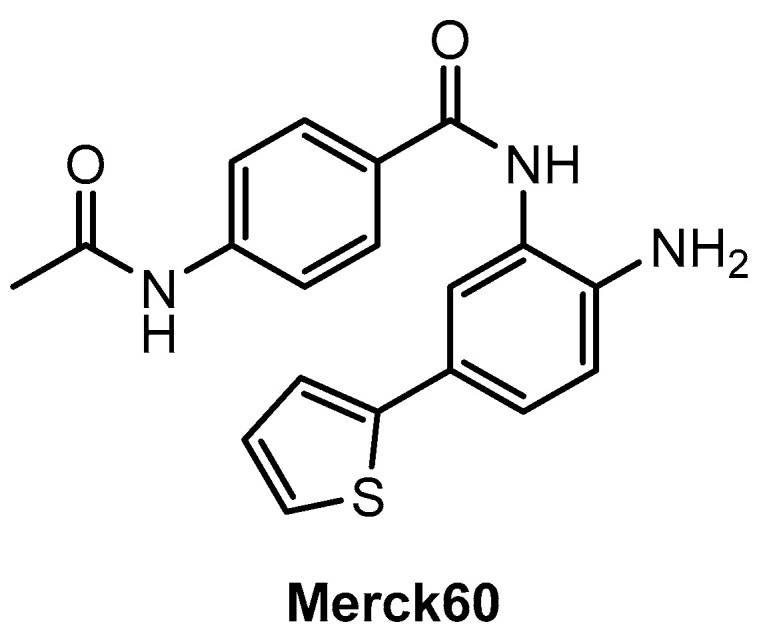
Chemical structure of Merck60.

Gryder, Wu, and co-workers [77] used the example of the benzamide-based compound “Merck60” (Figure 8) in their study. They demonstrated that an exchange of the thieno ring in the para-position of the benzamide core against a fluorine substituent in the meta-position can positively influence the selectivity for the isoenzyme HDAC3 compared to HDAC1/2. The reason for this can be explained in terms of the different sizes of the side pockets. It is smaller for HDAC3 than for HDAC1/2 and can be well targeted by the fluorine-substituted derivative, whereas the bulkier thieno ring does not favor this. Addressing smaller and hydrophobic binding pockets as a result of fluorine substitution is a common tool in medicinal chemistry [78]. The strategy has also been successfully applied, for example, in the design of fluorinated ligands, which exert their antitumor effect primarily through a more selective inhibition of the cyclooxygenase-2 (COX-2) enzyme, but not through influence on COX-1, unlike the corresponding methylated analogues [28,79].

**Figure 9:**
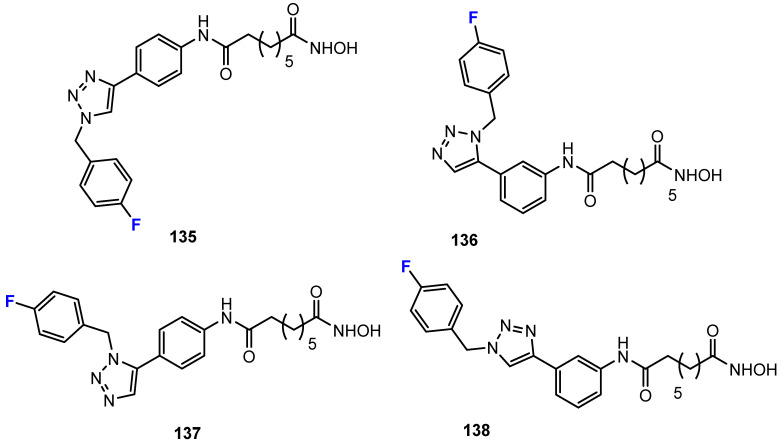
Chemical structures of fluorinated hydroxamic acids by Chen et al. [80,81].

In the series of triazolylphenyl-based hydroxamic acid derivatives (Figure 9) described as HDACis in the work of Chen et al. [80,81], fluorination of the phenyl ring in the para-position resulted in a doubling or tripling of the selectivity for the isoenzyme HDAC6 over HDAC1. Moreover, the introduction of a pentafluorophenyl cap in the study by Gawel, Shouksmith, Raouf, Nawar, and co-workers [71] was found to increase selectivity over HDAC6.

**Figure 10:**
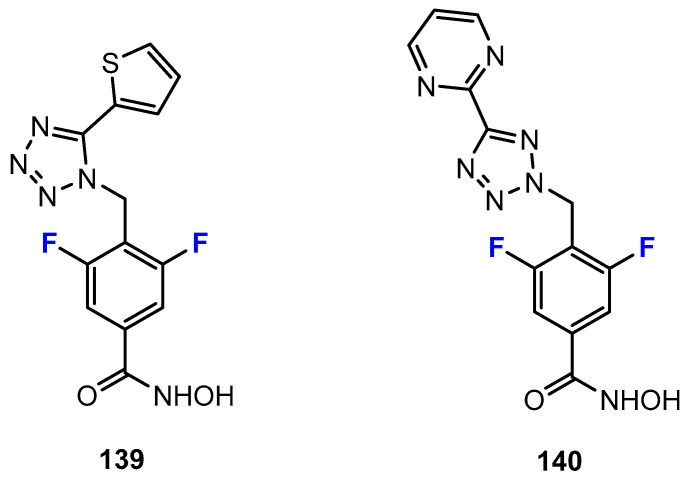
Chemical structure of fluorinated hydroxamic acids by Sandrone et al. [83].

The HDAC6 isoenzyme is of particular interest because its structure is somewhat different from the other isoenzymes [82]. Furthermore, the research work of Sandrone et al. [83] investigated the influence of fluorination on the selectivity towards the isoenzyme HDAC6. In a series of benzohydroxamate-based compounds (Figure 10), the linker in the form of the phenyl ring was single- or double-fluorinated. The effect on HDAC6 was not much improved, but the activity towards HDAC1 was decreased. This is ultimately accompanied by an increase in selectivity for HDAC6. Compared to the unfluorinated linkers, there is approximately a six-fold increase in selectivity for HDAC6 over HDAC1 after the introduction of two fluorine substituents. Specific interactions between the fluorine-containing linker and residues responsible in the binding pocket (i.e., Gly582, Ser531, and His614) of HDAC6 (HDAC class IIb) are suggested as the reason for this. In contrast, this is hindered in class I HDAC (e.g., HDAC1) because the Ser531 is exchanged by an Arg. These results of Sandrone et al. [83] were similarly confirmed in the study of Reßing, Schliehe-Diecks et al., where an increased inhibition of HDAC4 (HDAC class IIa) was detected due to compounds with a fluorinated linker [84].

Apparently opposed results were obtained from the fluorine scan undertaken by Zhang, Liu, Gao et al. [60,61] (Scheme 29). Fluorination on the thiolate residue of largazole derivatives revealed that the fluorinated compounds shifted in selectivity in favor of the isoenzyme HDAC1 relative to HDAC6.

The situation is again different for the 4-pentafluorosulfanyl-substituted vorinostat derivative. Compared to the parent compound, the activity towards HDAC6 is minimally increased. The introduction of the fluorine-containing substituent (Scheme 4) did not affect the inhibition profile of HDAC1 and HDAC2 [35]. This trend was also apparent with the trifluoromethylated congener [68].

Chuman et al. developed derivatives of vorinostat [55]. The hydroxamic acid residue, which serves as the zinc-binding site, was exchanged for a fluoroalkene containing a sulfanylmethyl group (Scheme 25). Additionally, the effects of the *E*- and *Z*-configurations on selectivity towards HDAC isozymes were investigated. In relation to the parent compound SAHA, there was no real change in the HDAC1/HDAC6 selectivity ratio. Nevertheless, the affinity of the *Z*-isomer towards the enzymes HDAC1, HDAC8, HDAC4, and HDAC6 was two to three times greater than with SAHA. The *E*-isomer had lower affinity, particularly for the HDAC1 and HDAC4 enzymes. However, these results do not necessarily indicate a shift in selectivity based on replacement by fluorine-containing substituents. The effects are more likely due to the *E/Z*-configuration. The authors considered this as a potential novel approach to designing isoform-selective HDACis.

**Figure 11:**
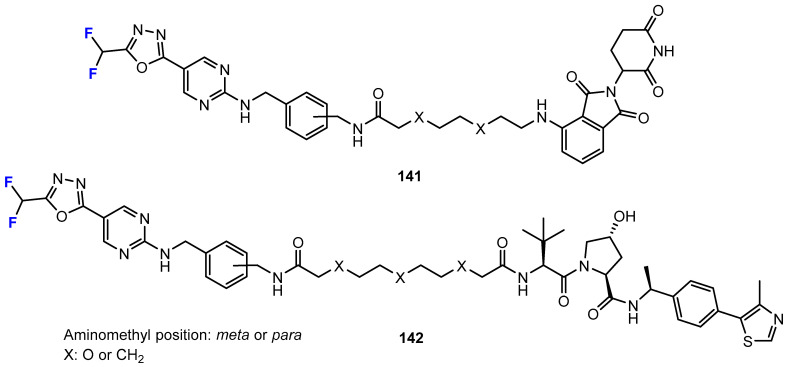
Chemical structures of fluorinated HDAC6-addressing PROTACs by Keuler, König, Bückreiß et al. [85].

The work of Keuler, König, Bückreiß, and colleagues [85] reports the development of compounds with a difluoromethyl-1,3,4-oxadiazole scaffold that serves to bind zinc instead of a hydroxamate (Figure 11). These non-hydroxamate compounds were found to function as proteolysis-targeting chimeras (PROTACs) for the intended degradation of a specific protein, e.g., HDAC. The compounds bearing the aminomethyl linker in the meta-position instead of the para-position, and with a spacer with oxyethylene subunits instead of pure alkyl groups, were found to be selective HDAC6 degraders compared to HDAC1 and HDAC4. Although the agents carry terminal conspicuous difluoromethyl groups, the influence of selectivity due to fluorination cannot be clearly deduced from this study.

In the paper by Ariawan et al. [42], the effect of vicinal difluorination in the chemical structure of the hydroxamate-based HDACis vorinostat and scriptaid was investigated (Scheme 11). The authors found that the introduction of fluorine substituents does not have a positive effect on the potency to inhibit HDAC. Among the vorinostat derivatives, there was little change in overall selectivity towards the eleven HDAC isoenzymes, as a result of fluorination. However, subtle effects on the selectivity of the class I HDACs could be detected. Finally, the authors concluded that it is rather challenging to engineer selective HDACis, especially when the binding pockets of the substrates (i.e., HDAC1, HDAC2, HDAC3) are narrow. This shows that fluorination alone is not necessarily crucial for bringing the substrate into the appropriate binding pocket of any isoenzyme. However, once it has reached the binding pocket, hydrophobic interactions formed by fluorine substituents can, of course, contribute to selectivity.

### 3.3. Labeling

The focus in medicinal chemistry is on designing new therapeutic agents through chemical synthesis. This development also includes the optimization of properties such as potency and selectivity. Furthermore, a major goal is understanding the influence of modifications in the chemical structure. Aside from the impact on the effect, there is interest in biodistribution, metabolism, or interaction with a target. In order to analyze the effectivity, it must be possible to sensitively detect the active agent in complex biological matrices. Fluorination is a good tracer for this purpose because fluorinated compounds rarely occur in nature. In addition, based on the isotopes ^19^F and ^18^F, it is possible to perform detection by spectroscopy and positron emission tomography (PET), respectively.

#### 3.3.1. Labeling Using ^19^F

**Figure 12:**
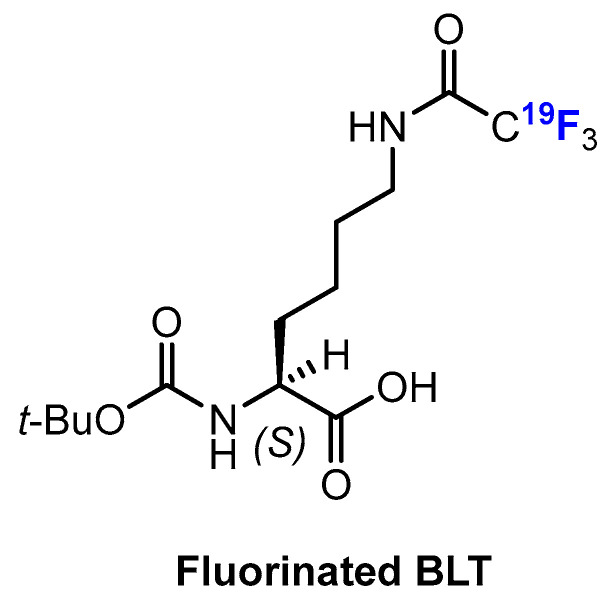
Chemical structure of fluorinated BLT by Sankaranarayanapillai et al. [86,87].

Sankaranarayanapillai and co-workers [86,87] developed a method based on NMR of ^19^F to monitor the inhibition of HDAC, since the fluorinated lysine derivative Boc-Lys-(Tfa)-OH (BLT) is a substrate for cleavage mediated by HDAC. Of note, treatment with BLT had no effect on the viability of PC3 cancer cells or on the activity of HDAC. Therefore, the amount of the ^19^F-labeled imaging agent BLT (Figure 12) detected by NMR spectroscopy allows conclusions to be drawn about the activity of HDAC. This method is non-invasive but does not rely on the fluorination of HDACis. Nevertheless, it has a distinct advantage. Since the treatment of a tumor with HDACis often leads to tumor stasis and not necessarily to its shrinkage, standard imaging methods would not necessarily be suitable to monitor this behavior. However, one drawback is that the different trifluoroacetyl-lysine-based substrates have different specificity with respect to HDAC isoenzymes; therefore, such substrates have limited applications [44].

In the study by Jayathilaka et al. [47], the introduction of a ^19^F tracer in the form of a trifluoromethyl group (Scheme 15) was used to gain insight into the mechanism of action of the known HDACi *N*-(2-aminophenyl)-*N’*-phenyloctanol diamine (BML-210). The activity of class IIa HDACs such as HDAC4 depends on the interaction with the Myocyte Enhancer Factor-2 (MEF2). Therefore, interference in this context represents a promising strategy to indirectly inhibit class IIa HDACs. The ^19^F-containing derivatives of BML-210 were used to study the interaction of the compound to bind MEF2 using NMR analysis. By comparing the ^19^F NMR spectrum of the free fluorinated compound with an approach in which the MEF2 protein was present in excess, it was possible to conclude that the drug interacts with MEF2.

Although these two examples represent the sophisticated use of ^19^F to characterize HDACis, NMR spectroscopy is inferior to PET in its prevalence and application.

#### 3.3.2. Labeling Using ^18^F

Labeling drugs with the radioisotope ^18^F is the most common modality of radiolabeling in both preclinical and clinical drug development [88]. There are a variety of chemical reactions, e.g., electrophilic or nucleophilic substitutions, to yield in radiofluorine-labeled drugs. Its importance is related to its accessibility by PET, as the isotope ^18^F undergoes β^+^ decay with a half-life of 109.7 min. By employing PET, it is possible to allow molecular imaging.

Probably the first HDAC imaging agent based on ^18^F-PET was developed by Hendricks, Keliher, and colleagues [44]. Compound **50** is a derivative of the clinically important HDACi vorinostat. The novel compound was obtained by introducing a radiofluorine substituent at position 4 of the aromatic ring of vorinostat (Scheme 13). With its low nanomolar potency, the compound showed a very similar biological profile to its parent compound. Using an ovarian cancer model in mice, the binding affinity to HDAC was investigated by ^18^F-PET. The authors found that quantification of the binding was possible up to 24 h after application. This paved the way for real-time in vivo imaging of HDACs, which the authors suggested will make a useful contribution to the characterization of HDACis.

Further studies include, for example, the development of radiofluorinated bioisosteres of the HDACi santacruzamate A as potential PET tracers [89]. Li and colleagues [52] introduced a [^18^F]fluoroethyl tracer into a biphenyl-based benzamide HDAC (i.e., INER-1577) to enable imaging (Scheme 23). They studied the distribution in the body and brain of rats using a micro-PET scanner. The compound was also able to inhibit the growth of breast cancer cell lines 4T1 and MCF-55. The authors continued synthesizing further benzamide-based HDACis with ^18^F tracers as imaging agents for mice [90]. The review by Dasko et al. [91] summarized further examples in which HDACis were fluorinated by the introduction of the ^18^F radiotracer, in order to study, among other things, the biodistribution and target binding of the compounds.

In their first study, Strebl et al. [45] used ^18^F labeling to assess the penetrance of the hydroxamic acid-based and non-fluorinated HDACi martinostat and the fluorinated derivative [^18^F]martinostat, respectively (Scheme 14). The radiofluorinated compounds showed promising properties as a radiotracer, as brain accumulation and distribution were similar to those of the ^11^C-labeled but non-fluorinated martinostat. However, the use of the ^19^F radioisotope instead of an ^11^C-labeled derivative (half-life ^11^C: 20.3 min) was associated with a longer half-life. In their following study [43], the authors prepared a radiofluorine version of the drug, i.e., [^18^F]bavarostat **47** (Scheme 12). Bavarostat belongs to the class of hydroxamic acid derivatives as HDAC inhibitors. The chemical structure of bavarostat also contains a fluorine substituent. However, it is assumed that the fluorine substituent does not affect the binding to HDAC [45]. Bavarostat itself is characterized by high selectivity to HDAC6. Since HDAC6 appears to play a role not only in cancer, but also in central nervous system diseases (e.g., Alzheimer’s disease), penetration into the brain is important, albeit difficult to reach with HDACis. The authors used the radiolabeled compound to determine the uptake into the brains of rodents and non-human primates. Bavarostat showed high penetration. Therefore, it was suggested that bavarostat is suitable for future investigations to target HDAC6 in living human brains.

**Figure 13:**
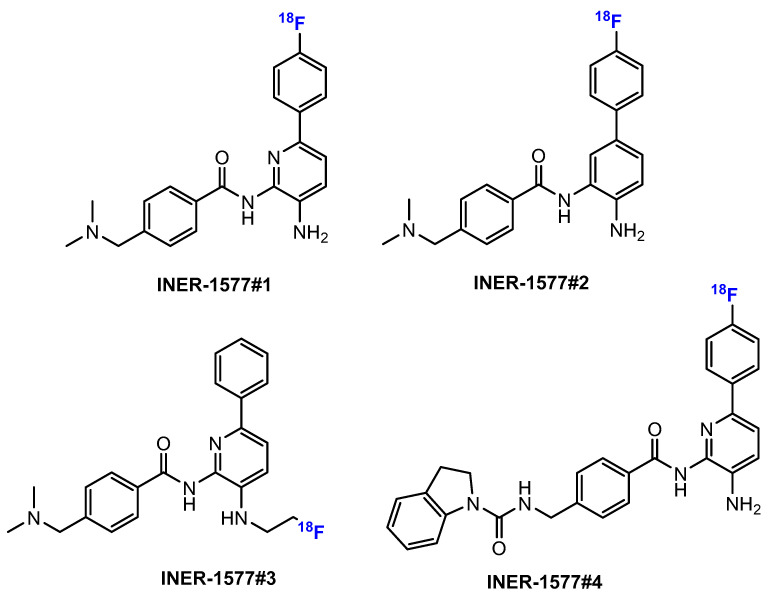
Chemical structures of four INER-1577 derivatives by Chen et al. [92].

The study by Chen et al. [92] also picked up four fluorinated HDACis designed for PET imaging due to their corresponding labeling with ^18^F (coded as INER-1577 #1 to INER-1577 #4, Figure 13). In particular, the compounds were again designed for Alzheimer’s disease detection, as HDACis are considered to play an important role in this context. The radiofluorinated HDACis serving as PET imaging compounds were investigated for their metabolism in biological systems, including a mass spectrometric approach. The authors concluded from their work that imaging studies should be performed promptly after application.

## 4. Issues of Fluorination

Even if fluorination can be beneficial for biological effects in terms of potency and selectivity or can be used in the context of labeling for bioanalytical studies, there are also some disadvantages.

The introduction of fluorine substituents into molecules is not always as trivial as it might seem and presents challenges to the medicinal chemist [93]. This may relate, for example, to the high toxicity or poor practical handling of fluorination reagents, as well as to low yields.

Among the HDACis mentioned by Luckhurst et al. [69], fluorine was also introduced in the form of difluoromethoxylation. The synthesis of difluoromethoxy derivatives is very commonly performed using chlorodifluoromethane and a base, following the elimination of HCl from chlorodifluoromethane. Chlorodifluoromethane is gaseous, and this synthetic procedure requires an ozone depleting reagent; these are major drawbacks of this approach [94]. On the other hand, the direct trifluoromethoxylation of aromatic compounds can be carried out under radical reactions, as described, for example, by Venturini et al. [95]. The gas trifluoromethyl hypofluorite is used. This particular reagent suffers from high toxicity [96]. Moreover, gases are generally more difficult to handle compared to solids. Even though liquids are easier to handle than gases, it can be considered a disadvantage to work with liquid reagents compared to solids as an alternative. The DexoFluor used for the synthesis of HDACis in the work of Ariawan et al. [42] is subject to this limitation to a certain extent. Fortunately, crystalline solids have now also been described as an improvement on DeoxoFluor for deoxyfluorination [97]. A different aspect is that if a C–F bond is to be introduced stereoselectively in the course of the synthesis, chiral auxiliaries can be used. This has the disadvantage that the auxiliary reagent must first be attached and then removed [98]. However, this is a general restriction and not limited to fluorine.

Examination of the series of HDACis by Meyners et al. [36] and Walton et al. [38] seems to reveal another issue. These are compounds that have a perfluorinated alkyl core. For compounds in which carbon atoms are completely substituted with halogens, photodissociation can occur under the release of the halogen into the stratosphere and the depletion of the ozone layer [97]. However, this effect seems to mainly apply to chlorine and bromine atoms rather than fluorine [99], but it would come into play when mixed halogenated alkanes are present. The formation of greenhouse gases is also mentioned in the review by Caron [97] as a disadvantage of the synthesis of fluorinated compounds. This environmental aspect is extended by the poor profile of fluorinating reagents with respect to green chemistry. In addition, the costs of the fluorinated reagents are very high in some cases, and there are safety concerns [97].

However, such concerns are not limited to the reagents used in the synthesis, but also affect the safety of drug use. Chemical instability and enzymes that metabolize active agents could lead to a loss of fluorination and the formation of metabolites that could probably affect the safety of therapy [100].

One of the main reasons why fluorination is used in medicinal chemistry drug design is its influence on metabolism. Since the dissociation energy of a C–F bond is very high, it seems that the generation of active metabolites can be avoided and the metabolic clearance can be decreased [100]. Nevertheless, a resealing of fluoride can occur. This can be seen by metabolizing enzymes or mediation by a nucleophile. If an agent undergoes a high cleavage of the C–F bond with the subsequent occurrence of relevant amounts of fluoride, skeletal fluorosis such as exostoses or periostitis may develop as adverse drug effects [101].

As comprehensively described by Pan [100], defluorination from monofluorinated alkyls bearing an intramolecular nucleophile can occur by an S_N_2 reaction. The nucleophile does not necessarily have to be present intramolecularly, but also in the form of biologically relevant compounds, such as glutathione. Furthermore, in the course of such an S_N_2 reaction, not only can fluoride be released, but also, biological targets can be alkylated, which could result in toxicity. Apart from the S_N_2 reaction, the heterolytic cleavage of a C–F bond yielding to the release of fluoride may also be due to a lone pair of electrons at the β-position of the fluorine [100]. The cleavage of fluoride can also be mediated by cytochrome P450 enzymes that catalyze the hydroxylation of fluorinated alkanes [100]. However, it is not only fluoride that leads to toxicological concerns, but also fluoroacetate [102]. This can be formed, for example, during the metabolism of molecules containing 1,3-difluoro-2-propyl or O-/N-2-fluoroethyl substituents [100].

## 5. Conclusions

Currently, HDACs increasingly prove to play an important role in cancer via chromatin-modifying complexes and modulating gene expression [10]. Therefore, these enzymes become potential and attractive targets in the research and development of new anticancer drugs [9]. As a result, many HDACis have been discovered and studied in recent years. One of the strategies for the design of novel pharmaceuticals is to introduce fluorine into the chemical structures [103]. This approach has also been performed with HDACis such as hydroxamic acids, benzamides, thiols, or cyclic peptides. However, no overview is available on this aspect so far in the literature to the best of our knowledge.

Fluorination has been shown to have many advantages, as described above in this review. These include partly higher potency, increased binding affinity, and expanded selectivity towards the different HDAC isoenzymes [44]. Moreover, altered physicochemical properties and improved metabolic stability can also be attributed to the introduction of fluorine 92. Yet, it should also be noted that chemical instability and enzymes that metabolize active agents could lead to a loss of fluorination and the formation of metabolites that could probably affect the safety of drug use [104]. Nevertheless, chemical instability and metabolizing enzymes (e.g., CYP enzymes) could cause a loss of fluorination and the generation of likely toxic metabolites that compromise drug safety [100].

In the field of radiation oncology, fluorine-18 (^18^F) represents a radioisotope which is one of the most common isotopes used for PET imaging [105]. It has a half-life of approximately 110 min, more than five times longer compared to the half-life of carbon-11 (approximately 20 min) [44].

This review summarized several HDACis-related studies focusing on the synthesis, as well as the purposes and influences, of fluorination developed in recent years. The development of synthetic methods for the formation of fluorinated derivatives and the advantages of incorporating fluorine into the chemical scaffold of new HDACis will certainly open new opportunities in the research and development of anticancer agents in the future.

## Data Availability

Data is contained within the article.
